# Influence of Type of Dental Visit on the Incidence of COVID-19 and Related Hospitalisation Among Older People in Japan

**DOI:** 10.3390/ijerph21121668

**Published:** 2024-12-14

**Authors:** Mizuki Saito, Yoshihiro Shimazaki, Toshiya Nonoyama, Yoshinori Inamoto

**Affiliations:** 1 Department of Preventive Dentistry and Dental Public Health, School of Dentistry, Aichi Gakuin University, Nagoya 464-8650, Japan; saito@dpc.agu.ac.jp (M.S.); ag113d15@dpc.agu.ac.jp (T.N.); 2 Mie Dental Association, Tsu 514-0003, Japan; y-ina@nifty.com

**Keywords:** COVID-19, dental visit, hospitalisation, older people, periodontal treatment

## Abstract

In 2020, the coronavirus disease 2019 (COVID-19) pandemic began worldwide. We examined the association between dental visit status and the incidence of COVID-19 and hospitalisation for it among older people based on medical claims data to help reduce COVID-19 severity. The study included 170,232 people who were 75–85 years old in fiscal 2019, with fiscal 2020 and 2021 serving as the follow-up period to ascertain the status of COVID-19. Using medical claims data, we investigated four types of dental visit (no visit, only periodontal treatment, periodontal and other treatment, and only other treatment) during fiscal 2019 and the incidence of COVID-19 and hospitalisation for COVID-19 during the follow-up period. Logistic regression analyses were performed with the incidence of COVID-19 and hospitalisation for COVID-19 as the dependent variables. Of the participants, 3206 (1.9%) developed COVID-19, of whom, 559 (17.4%) were hospitalised. There was not a significant association between the incidence of COVID-19 and type of dental visit. Participants with dental visits for periodontal treatment during the baseline year had a significantly lower odds ratio (OR) for hospitalisation due to COVID-19 compared to those without dental visits (OR: 0.71, 95% confidence interval: 0.58–0.78). The results suggest that dental visits for periodontal treatment including maintenance are important not only for maintaining oral health but also for preventing severe COVID-19.

## 1. Introduction

An outbreak of pneumonia due to an unknown cause in Wuhan, Hubei Province, China, in December 2019 was later found to be caused by a novel coronavirus, SARS-CoV-2, which is an animal-derived beta coronavirus; the same pathogen is responsible for severe acute respiratory syndrome (SARS) and Middle East respiratory syndrome (MERS) [1]. Subsequently, coronavirus disease 2019 (COVID-19) spread worldwide via human-to-human SARS-CoV-2 infection.

Although the number of new cases and deaths from COVID-19 is now declining, vigilance is still needed because of increases in some areas and the possibility of virus mutation. The severity of COVID-19 among older people has decreased significantly from its peak but is still high compared to younger patients [2]. In addition, worsening of the underlying disease and a reduction in activities of daily living due to hospitalisation significantly affect the quality of life and life expectancy of older people [3]. Recently, an increasing number of people have been suffering from post-COVID-19, a condition in which symptoms persist after contracting COVID-19 [4]. Post-COVID-19 has a variety of symptoms, many of which improve over time. For some, however, symptoms do not improve and can hinder their return to society and reduce their quality of life [4]. Severe COVID-19 is a risk factor for the developing post-COVID-19 [5]. Therefore, it is important to identify factors affecting the incidence of COVID-19 and COVID-19 severity in older people.

There have been many reports on the association between oral health and COVID-19 [6,7,8,9,10,11,12,13,14,15,16,17]. The COVID-19 pandemic affected the use of dental services [13,14,16], with an increase in emergency visits and a decline in routine oral health services [13]. Thus, the COVID-19 pandemic worsened the oral health status of the population [18]. Oral health status has been suggested to influence the severity of COVID-19 [6,7,8,9,10,11,12,13,15,17]. An association between periodontal disease and COVID-19 has been reported [7,10,11,12,13,15,17]. In a case–control study of COVID-19 patients, those with periodontitis had a significantly higher risk of COVID-19 complications, including intensive care unit admissions, use of assisted ventilation, and death, than those without periodontitis [10]. In another study, COVID-19 patients with periodontitis were at higher risk of hospital admission and requiring assisted ventilation [12]. Since periodontal disease may be a risk for more severe COVID-19, maintaining good periodontal status, including through dental visits, may help to reduce the severity of COVID-19. Older people are at high risk for periodontitis, tooth loss, and oral functional decline and require regular management and appropriate treatment at dental offices [19]. However, no study has examined the association between dental visits and the incidence of COVID-19 or hospitalisation due to COVID-19.

In this study, we used medical claims data to examine associations between types of dental visit and the incidence of COVID-19 and related hospitalisation in older people in Japan. The main goal was to maintain the quality of life in older people by preventing severe COVID-19.

## 2. Materials and Methods

### 2.1. Study Participants

In Japan, people aged 75 and older are required to enrol in the late-stage medical care system. This study included those insured under the late-stage medical care system in Mie Prefecture who were 75–85 years old in fiscal 2019, the baseline year. This age range was chosen because data on people in this age group were provided by the insurer. The study participants included those without medical claims. Those who were no longer insured between 2019 and 2021 due to death or relocation were excluded. Fiscal 2020 and 2021 served as the follow-up period to ascertain the status of COVID-19. Using medical claims data from the Mie Prefecture late-stage medical care system, we investigated the incidence of COVID-19 and hospitalisation for COVID-19 in fiscal 2020–2021. Because of the inability to determine the cause of death, participants who died during the period 2019–2021 were excluded from the analysis.

### 2.2. Type of Dental Visits

In the Japanese social insurance system, dental diseases are classified into three categories based on the International Statistical Classification of Diseases and Related Health Problems 10th Revision (ICD-10): dental caries (ICD-10 codes: K00-K04), periodontal disease (ICD-10 code: K05), and other disorders of the teeth or supporting tissues of teeth (ICD-10 code: K06-K08). In this study, the participants were classified into four groups: the types of dental visit based on the presence or absence of dental visits in the three disease categories. Because periodontal disease is commonly used to classify patients undergoing regular management in Japan, the classification of type of dental visit focused on periodontal disease. Of those who made a visit for any dental disease, those who made a visit for periodontal disease only were classified as periodontal treatment; those who made a visit for periodontal disease and dental caries or other disease were classified as periodontal treatment and other treatment; and those who made no visit for periodontal treatment were classified as other treatment. Those who made no visit for any dental disease were defined as no visit.

### 2.3. COVID-19

Since the beginning of the COVID-19 pandemic, the World Health Organization has been adding emergency codes for COVID-19 to the ICD-10. Emergency codes were initially added for COVID-19 (codes U07.1 and U07.2) in February 2020. Moreover, a personal history of COVID-19 (code U08), post-COVID-19 condition (code U09), and multisystem inflammatory syndrome associated with COVID-19 (code U10) were added in September 2020, and codes for immunisation to prevent COVID-19 (code U11) and adverse reaction to a COVID-19 vaccine (code U12) were added in January 2021.

In the medical claims data used in this study, ICD10 codes U06–U49 were classified as emergency codes. Therefore, we did not identify only COVID-19 (codes U07.1 and U07.2). However, since Zika virus infection (code U06) has not been confirmed in Japan to date, codes U13–U49 do not have specific names at this time, and codes U08–U12 are COVID-19-related. Therefore, participants with codes U06–U49 in the insurance claims database were considered to have a history of COVID-19. We then determined whether participants had any medical visits associated with COVID-19 or hospitalisation due to COVID-19 during the period 1 April 2020 to 31 March 2022.

### 2.4. Confounding Factors

Total medical costs for fiscal 2019 at baseline were calculated from the medical claims data and used as an indicator of the participants’ overall health status. Diabetes mellitus, asthma, chronic obstructive pulmonary disease (COPD), stroke, and ischemic heart disease are considered risk factors for severe coronary disease. The presence or absence of medical visits for those diseases was calculated from medical claims data (ICD-10 code; diabetes mellitus: E10–E14, asthma: J45–J46, COPD: J41–J44, stroke: I63 and I69.3, and ischemic heart disease: I20–I25). Under the Japanese healthcare system, annual income determines the insurance premiums and limits of co-payments. One of the criteria used is regardless of whether the resident tax is levied. Those whose annual income is below a certain level are exempt from the resident tax, and their premiums and limits are set lower. In this study, the presence or absence of resident taxation included in the medical claims data was used as economic status, with those who were exempt from resident taxation defined as low income and those who were taxed defined as normal income. The criterion for resident taxation is an annual household income of at least JPY 1.48 million.

### 2.5. Statistical Analysis

We analysed the association between type of dental visit during the baseline year and the incidence of COVID-19 and hospitalisation due to COVID-19 during the follow-up year. The association between each variable and COVID-19 incidence and hospitalisation and the association between each variable and type of dental visit were analysed using the analysis of variance (for comparison of means) and the chi-square test (for comparison of proportions). Logistic regression analysis was performed with COVID-19 incidence as the dependent variable and age, sex, economic status, medical history, total medical costs, and type of dental visit as independent variables, and odds ratios (ORs) and 95% confidence intervals (CIs) were determined. To examine the association between dental visits and the severity of COVID-19, logistic regression analysis was performed with hospitalisation due to COVID-19 as the dependent variable among participants with COVID-19 onset in fiscal 2020 and 2021. The adequacy of the sample size in the multivariate logistic regression analysis was confirmed by the number of minority categories in the dependent variable being at least 10-fold the number of independent variables used in the analysis [20]. All statistical analyses were performed using SPSS software (ver. 28.0; IBM Corp., Armonk, NY, USA). A *p*-value < 0.05 was considered to indicate statistical significance.

## 3. Results

**Figure 1:**
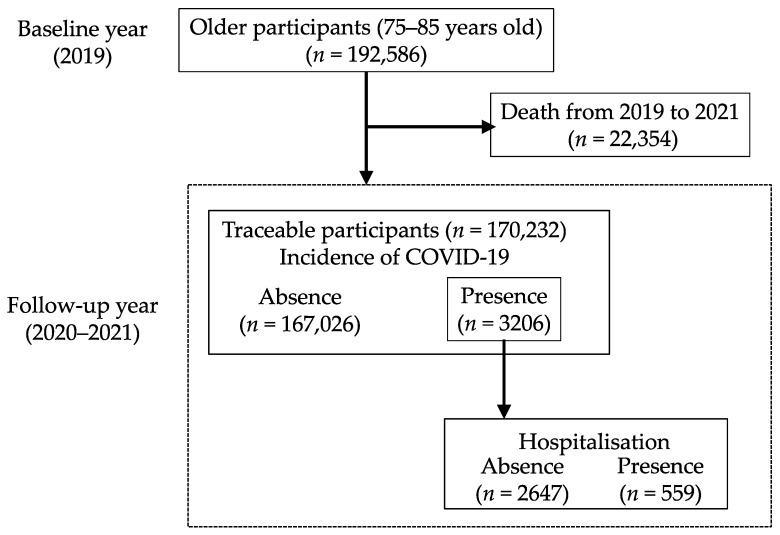
Flow-chart of study participant selection.

Of the 192,586 participants aged 75–85 years in fiscal 2019, 170,232 were included in the analysis, and 22,354 who died in 2019–2021 were excluded (Figure 1). Of the participants, 3206 (1.9%) developed COVID-19 during fiscal 2020–2021, of whom, 559 (17.4%) were hospitalised due to COVID-19.

Table 1 shows the associations between each variable and the type of dental visit. Among participants, 78,601 (46.2%) had no dental visit, 64,315 (37.8%) received only periodontal treatment, 15,549 (9.1%) received periodontal and other treatment, and 11,767 (6.9%) received only other treatment. Age, sex, total medical cost, economic status, and medical history of diabetes mellitus, asthma, COPD, stroke, and ischemic heart disease were significantly associated with type of dental visit.

Table 2 shows the results of the univariate and multivariate logistic regression analyses with the incidence of COVID-19 as the dependent variable. Age, economic status, and medical history of asthma, stroke, and ischemic heart disease were significantly associated with COVID-19 incidence in multivariate analyses. There were no significant associations between the incidence of COVID-19 and type of dental visit.

Table 3 shows the results of univariate and multivariate logistic regression analyses with hospitalisation due to COVID-19 as the dependent variable. Age, total medical costs, and dental visits were significantly associated with hospitalisation due to COVID-19 in the multivariate analysis. Participants with dental visits for periodontal treatment during the baseline year had a significantly lower OR for hospitalisation due to COVID-19 than those without dental visits. The OR for those who received only periodontal treatment was 0.71 (95% CI: 0.58–0.87), and that for those who received periodontal and other treatment was 0.67 (95% CI: 0.46–0.96).

## 4. Discussion

There were no significant differences in the incidence of COVID-19 among the types of dental visit before the COVID-19 epidemic, but those who had dental visits for periodontal treatment had a significantly lower risk of hospitalisation due to COVID-19.

In Japan, those aged 75 or older are required to enrol in the medical insurance system, with the exception of welfare recipients. The data used in this study were such medical claims data from the Mie Prefecture medical insurance system (the welfare receipt rate among persons 65 years and older in Mie Prefecture in 2019 was 1.57%). The reliability and value of the study results are considered high because of the large number of people surveyed and the small degree of selection bias. Our results are valuable because there are no reports on the influence of dental visits on the incidence and severity of COVID-19. The fact that this is a cohort study is a study strength, as most studies on the association between COVID-19 and oral health have been cross-sectional or case–control studies [13,15].

Nevertheless, there are numerous limitations to this study. The greatest is that only medical claims data were used. Consequently, the actual oral status and dental treatment were not known. Participants who had dental visits for periodontal treatment were considered to have good oral health due to maintenance at a dental clinic, but they included some with severe periodontitis. However, smoking habit and systemic diseases such as obesity, diabetes mellitus, and lung disease, which are risk factors for COVID-19 [21], were not considered. An important limitation of this study is that, although it was a longitudinal study, a causal relationship between the type of dental visit and the severity of COVID-19 could not be determined, nor was it possible to infer the mechanism underlying the relationship. As another limitation, the results are not generalisable to younger people or to older people in other regions, because the data were obtained from older people in one prefecture in Japan. Because the cause of death could not be determined, even if the participants died of COVID-19 in fiscal 2020 and 2021, they were excluded from the study. People who died or moved during the study period were also excluded from the analysis, which may have introduced attrition bias. During the study period, COVID-19 was an ongoing pandemic, such that some patients who should have been hospitalised had difficulty being admitted [22]. Since details on the condition of patients hospitalised with COVID-19 were unavailable, the proportion who were in a serious condition was unknown. However, it is reasonable to assume that COVID-19 patients who visited medical institutions and were subsequently hospitalised included patients who were more severity ill than those who were not hospitalised. The dental visits considered to affect COVID-19 in this study were in fiscal 2019, before the epidemic. Dental visits in fiscal 2020 and 2021, when COVID-19 infection was confirmed, were not considered because they were considered to differ from health behaviours in normal periods, as many people tended to refrain from non-urgent dental visits during the outbreak [18].

It has been reported that periodontal disease is associated with severe COVID-19 [8,10,12]. In a case–control study of COVID-19 patients, those with moderate to severe periodontitis had significantly higher ORs for COVID-19 complications, including intensive care unit admissions, assisted ventilation, and death, than those with healthy periodontium or mild periodontitis [10]. Those with painful and bleeding gums have a higher mortality rate due to COVID-19 [7], and periodontal disease may exacerbate the effect of obesity on hospitalisation and mortality following COVID-19 infection [17]. In a study of the association between oral health status and COVID-19, a lack of occlusal support was a risk for intensive care unit admission in COVID-19 patients [11]. However, another study reported that there was no association between tooth loss and mortality and hospitalisation for COVID-19 [7].

Several studies have examined the association between dental visits and COVID-19 [13,14,16]. A study on the use of dental services during the COVID-19 pandemic reported an increase in the utilisation of emergency dental visits and a decline in the utilisation of routine oral health services [14,16]. A study of Japanese office workers showed that individuals who discontinued regular dental visits during the COVID-19 pandemic had relatively poor periodontal health [18]. However, there are no reports on the influence of dental visits on the incidence and severity of COVID-19.

The mechanism by which oral health status affects COVID-19 severity is not clear, but chronic inflammatory conditions due to periodontal disease may affect COVID-19 severity [23]. Cytokine storms are associated with COVID-19 severity [24]. A key mediator in the process leading to a cytokine storm is interleukin (IL)-6, which activates the IL-6 amplifier, leading to the production of high levels of inflammatory cytokines and chemokines [25,26]. The presence of chronic inflammation, as seen in periodontal disease, for example, may increase IL-6 amplifier activation [27], and older adults without dental visits may be more prone to IL-6 amplifier activation due to periodontal disease. Older people are at high risk for aspiration pneumonia due to decreased swallowing function [28]. Therefore, the risk of coinfection of SARS-CoV-2 and oral bacteria in the lungs is increased in older people. Periodontal pathogens such as Capnocytophaga and *Veillonella*, and other oral opportunistic pathogens, were detected in bronchoalveolar lavage fluid from COVID-19 patients [29]. Therefore, it is possible that those who had dental visits for periodontal treatment at baseline were less likely to have severe COVID-19 because they might had better oral health.

However, based on the results of this study, it is difficult to demonstrate a preventive effect of dental visits on the severity of COVID-19. The most significant factor in the association between dental visits and COVID-19 severity may be the influence of systemic health status at baseline. Those who had dental visits were considered to be in relatively good health. Indeed, people in good health are at lower risk of developing severe COVID-19 [30]. On the other hand, those with impaired physical function and those suffering from diseases such as diabetes have been reported to have lower rates of dental visits than healthy individuals [31]. Thus, in this study, those who did not have a dental visit were in poorer systemic health to begin with, which may have contributed to the severity of COVID-19. Histories of diabetes mellitus, respiratory disease, and cardiovascular disease, which are considered risk factors for severe COVID-19, were considered confounding factors in the analysis based on the presence or absence of medical visits for these diseases during the baseline year. However, because the determination of the presence of systemic diseases was based solely on medical claims data, it does not accurately reflect actual health status as it does not capture the severity of the diseases or the details of medical treatment, thus limiting its ability to adjust actual health status. In addition, those who visit dental clinics regularly have higher health awareness and better lifestyle habits, such as exercise and not smoking, than those who do not habitually make dental visits [32], which may contribute to the lower risk of severe COVID-19. Thus, the difference in the risk of COVID-19 severity between those with and without dental visits may be due to the influence of differences in health status and lifestyle.

In this study, dental visits were classified into four types: no visit, only periodontal treatment, periodontal and other treatment, and only other treatment. Patients in the only periodontal treatment group presumably included those who received either basic or more advanced periodontal treatment, as well as those who received regular periodontal maintenance. The periodontal and other treatment group were assumed to comprise participants who received periodontal treatment, regular maintenance, and other restorative or prosthetic treatment. The difference between the two groups would have depended on the dental condition of each patient, but distinguishing between the two groups was difficult because the specific oral condition of the respective participants was unknown. The group of only other treatment visited a dentist but did not receive periodontal treatment. Participants who receive periodontal treatment may not have good oral health. However, since the proportion of people visiting a dentist for maintenance after periodontal treatment is increasing in Japan [33], the participants who visited a dentist for periodontal treatment may have had a relatively high awareness of their oral health. While this was a limitation of the real-world data used in this study, our results nonetheless suggest that the type of dental visit reflects the participant’s overall health condition.

In a study of the association between the use of dental services and health outcomes, those who received preventive dental care were less likely to be hospitalised or use emergency services [34]. Thus, the possibility that dental visits have a positive effect on reducing the severity of COVID-19 is not entirely ruled out, even after considering these limitations, because dental visits have been reported to maintain overall health. To clarify whether dental visits and maintaining oral health can reduce the severity of infectious diseases such as COVID-19, it is necessary to analyse the relationship between dental visits and actual oral conditions and severe COVID-19, fully considering the risk of severe COVID-19, such as health status and lifestyle habits.

## 5. Conclusions

There were no significant differences in the incidence of COVID-19 among the types of dental visit, but the risk of severe COVID-19 was lower in those with dental visits for periodontal treatment. For older people, dental visits for periodontal treatment and regular maintenance may help maintain their oral health, which may also be beneficial for their overall health.

## Figures and Tables

**Table 1:** Associations of the characteristics of the participants with the type of dental visit.

	Type of Dental Visit				
	No Visit (*n* = 78,601)	Only Periodontal Treatment (*n* = 64,315)	Periodontal and Other Treatment (*n* = 15,549)	Only Other Treatment (*n* = 11,767)	*p*-Value
	Mean (SD)				
Age (years)	79.7 (3.2)	78.8 (3.0)	78.9 (3.0)	79.5 (3.1)	<0.001
Total medical costs (2019) (×JPY 1000)	48,236.9 (105,857.0)	45,584.1 (93,142.3)	50,229.7 (102,546.4)	50,500.0 (101,246.3)	<0.001
	*n* (%)				
Sex					
Men	31,592 (40.2)	27,241 (42.4)	6960 (44.8)	5292 (45.0)	<0.001
Women	47,009 (59.8)	37,074 (57.6)	8589 (55.2)	6475 (55.0)	
Economic status					
Normal	49,452 (62.9)	45,986 (71.5)	11,009 (70.8)	7753 (65.9)	<0.001
Low	29,149 (37.1)	18,329 (28.5)	4540 (29.2)	4014 (34.1)	
Medical history					
Diabetes mellitus					
No	69,682 (88.7)	57,362 (89.2)	13,678 (88.0)	10,313 (87.6)	<0.001
Yes	8919 (11.3)	6953 (10.8)	1871 (12.0)	1454 (12.4)	
Asthma					
No	77,061 (98.0)	62,759 (97.6)	15,158 (97.5)	11,482 (97.6)	<0.001
Yes	1540 (2.0)	1556 (2.4)	391 (2.5)	285 (2.4)	
COPD					
No	77,536 (98.6)	63,507 (98.7)	15,319 (98.5)	11,576 (98.4)	0.005
Yes	1065 (1.4)	808 (1.3)	230 (1.5)	191 (1.6)	
Stroke					
No	74,425 (94.7)	60,943 (94.8)	14,637 (94.1)	11,076 (94.1)	0.001
Yes	4176 (5.3)	3372 (5.2)	912 (5.9)	691 (5.9)	
Ischemic heart disease					
No	74,878 (95.3)	60,870 (94.6)	14,567 (93.7)	11,082 (94.2)	<0.001
Yes	3723 (4.7)	3445 (5.4)	982 (6.3)	685 (5.8)	
Incidence of COVID-19					
Absence	77,120 (98.1)	63,107 (98.1)	15,275 (98.2)	11,524 (97.9)	0.348
Presence	1481 (1.9)	1208 (1.9)	274 (1.78)	243 (2.1)	

Note: COVID-2019, coronavirus disease 2019; SD, standard deviation; JPY, Japanese yen; COPD, chronic obstructive pulmonary disease.

**Table 2:** Univariate and multivariate logistic regression analyses of the associations between the independent variables and the incidence of COVID-19.

	Incidence of COVID-19		Dependent Variable: Incidence of COVID-19 (Absence = 0, Presence = 1)			
Independent Variable	Absence (*n* = 167,026)	Presence (*n* = 3206)	Crude OR (95% CI)	*p*-Value	Adjusted OR (95% CI)	*p*-Value
	Mean (SD)					
Age (years)	79.3 (3.1)	79.5 (3.2)	1.03 (1.01–1.04)	<0.001	1.03 (1.02–1.04)	<0.001
Total medical costs (2019) (×JPY 1000)	47,505.5 (100,639.7)	51,096.9 (99,307.4)	1.00 (1.00–1.00)	0.045	1.00 (1.00–1.00)	0.169
	*n* (%)					
Sex						
Men	69,695 (98.0)	1390 (2.0)	1		1	
Women	97,331 (98.2)	1816 (1.8)	0.94 (0.87–1.00)	0.064	0.97 (0.91–1.05)	0.472
Economic status						
Normal	111,929 (98.0)	2271 (2.0)	1		1	
Low	55,097 (98.3)	935 (1.7)	0.84 (0.78–0.90)	<0.001	0.83 (0.77–0.90)	<0.001
Medical history						
Diabetes mellitus						
No	148,218 (98.1)	2817 (1.9)	1		1	
Yes	18,808 (98.0)	389 (2.0)	1.09 (0.98–1.21)	0.122	1.08 (0.97–1.20)	0.165
Asthma						
No	163,342 (98.1)	3118 (1.9)	1		1	
Yes	3684 (97.7)	88 (2.3)	1.25 (1.01–1.55)	0.040	1.26 (1.01–1.56)	0.037
COPD						
No	164,777 (98.1)	3161 (1.9)	1		1	
Yes	2249 (98.0)	45 (2.0)	1.04 (0.78–1.40)	0.781	1.01 (0.75–1.36)	0.939
Stroke						
No	158,089 (98.1)	2992 (1.9)	1		1	
Yes	8937 (97.7)	214 (2.3)	1.27 (1.10–1.46)	0.001	1.24 (1.08–1.43)	0.003
Ischemic heart disease						
No	158,385 (98.1)	3012 (1.9)	1		1	
Yes	8641 (97.8)	194 (2.2)	1.18 (1.02–1.37)	0.027	1.15 (0.99–1.33)	0.066
Type of dental visit						
No visit	77,120 (98.1)	1481 (1.9)	1		1	
Only periodontal treatment	63,107 (98.1)	1208 (1.9)	1.00 (0.92–1.08)	0.934	1.00 (0.93–1.08)	0.921
Periodontal and other treatment	15,275 (98.2)	274 (1.8)	0.93 (0.82–1.06)	0.304	0.93 (0.82–1.06)	0.302
Only other treatment	11,524 (97.9)	243 (2.1)	1.10 (0.96–1.26)	0.181	1.09 (0.95–1.25)	0.213

Note: COVID-2019, coronavirus disease 2019; OR, odds ratio; CI, confidence interval; SD, standard deviation; JPY, Japanese yen; COPD, chronic obstructive pulmonary disease.

**Table 3:** Univariate and multivariate logistic regression analyses of the associations between the independent variables and hospitalisation due to COVID-19.

	Hospitalisation Due to COVID-19		Dependent Variable: Hospitalisation Due to COVID-19 (Absence = 0, Presence = 1)			
Independent Variable	Absence (*n* = 2647)	Presence (*n* = 559)	Crude OR (95% CI)	*p*-Value	Adjusted OR (95% CI)	*p*-Value
	Mean (SD)					
Age (years)	79.4 (3.2)	80.1 (3.1)	1.08 (1.05–1.11)	<0.001	1.07 (1.04–1.10)	<0.001
Total medical costs (2019) (×JPY 1000)	48,461.3 (94,050.0)	63,577.1 (120,462.0)	1.00 (1.00–1.00)	0.001	1.00 (1.00–1.00)	0.002
Sex	*n* (%)					
Men	1139 (81.9)	251 (18.1)	1		1	
Women	1508 (83.0)	308 (17.0)	0.93 (0.77–1.11)	0.417	0.86 (0.71–1.04)	0.127
Economic status						
Normal	1889 (83.2)	382 (16.8)	1		1	
Low	758 (81.1)	177 (18.9)	1.16 (0.95–1.41)	0.153	1.13 (0.92–1.38)	0.259
Medical history						
Diabetes mellitus						
No	2332 (82.8)	485 (17.2)	1		1	
Yes	315 (81.0)	74 (19.0)	1.13 (0.86–1.48)	0.379	1.13 (0.86–1.49)	0.384
Asthma						
No	2578 (82.7)	540 (17.3)	1		1	
Yes	69 (78.4)	19 (21.6)	1.32 (0.79–2.20)	0.299	1.30 (0.77–2.19)	0.329
COPD						
No	2613 (82.7)	548 (17.3)	1		1	
Yes	34 (75.6)	11 (24.4)	1.54 (0.78–3.06)	0.216	1.51 (0.75–3.03)	0.245
Stroke						
No	2467 (82.5)	525 (17.5)	1		1	
Yes	180 (84.1)	34 (15.9)	0.89 (0.61–1.30)	0.537	0.80 (0.55–1.18)	0.268
Ischemic heart disease						
No	2486 (82.5)	526 (17.5)	1		1	
Yes	161 (83.0)	33 (17.0)	0.97 (0.66–1.43)	0.872	0.88 (0.59–1.30)	0.510
Type of dental visit						
No visit	1180 (79.7)	301 (20.3)	1		1	
Only periodontal treatment	1034 (85.6)	174 (14.4)	0.66 (0.54–0.81)	<0.001	0.71 (0.58–0.87)	<0.001
Periodontal and other treatment	236 (86.1)	38 (13.9)	0.63 (0.44–0.91)	0.014	0.67 (0.46–0.96)	0.031
Only other treatment	197 (81.1)	46 (18.9)	0.92 (0.65–1.29)	0.616	0.93 (0.66–1.32)	0.677

Note: COVID-2019, coronavirus disease 2019; OR, odds ratio; CI, confidence interval; SD, standard deviation; JPY, Japanese yen; COPD, chronic obstructive pulmonary disease.

## Data Availability

The data that support the findings of this study are available from the wide-area union in Mie Prefecture. Restrictions apply to the availability of these data, which were used under licence for this study. Data are available from the authors with the permission of the wide-area union in Mie Prefecture.
